# Tungiasis—A Neglected Disease with Many Challenges for Global Public Health

**DOI:** 10.1371/journal.pntd.0003133

**Published:** 2014-10-30

**Authors:** Hermann Feldmeier, Jorg Heukelbach, Uade Samuel Ugbomoiko, Elizabeth Sentongo, Pamela Mbabazi, Georg von Samson-Himmelstjerna, Ingela Krantz, The International Expert Group for Tungiasis

**Affiliations:** 1 Institute of Microbiology and Hygiene, Campus Benjamin Franklin, Charité University Medicine, Berlin, Germany; 2 Department of Community Health, School of Medicine, Federal University of Ceará, Fortaleza, Brazil; 3 Department of Zoology, University of Ilorin, Kwara State, Illorin, Nigeria; 4 Makerere University College of Health Sciences, School of Biomedical Sciences, Department of Medical Microbiology, Kampala, Uganda; 5 World Health Organization, Geneva, Switzerland; 6 Institute for Parasitology and Tropical Veterinary Medicine, Faculty of Veterinary Medicine, Freie Universität, Berlin, Germany; 7 Skaraborg Institute for Research and Development, Skövde, Sweden

## Introduction

Tungiasis (sand flea disease) is a parasitic skin disease with origins in South America. It was introduced into sub-Saharan Africa in the 19th century [1]–[3]. Sand flea disease is a zoonosis caused by the penetration of female sand fleas into the skin. In humans, tungiasis predominantly affects marginalized populations. Children and elderly people are especially susceptible to severe disease. Sand flea disease is the most frequent parasitic infection in many resource-poor communities. In animals like dogs, pigs, or ruminants, the infection has severe consequences with, for example, reduced milk production when the skin of the udder is affected.

Despite the substantial disease burden caused by embedded sand fleas, tungiasis is basically neglected by health care providers, policy makers, the scientific community, the pharmaceutical industry, and funding institutions [4]. Although not included in WHO's list of neglected tropical diseases (NTDs), tungiasis bears all the hallmarks of an NTD to merit apprehension from the public health sector [5]–[7]. It occurs in resource-poor communities, causing considerable morbidity and loss of quality of life. Systematic data on disease occurrence are not available.

## Medical History

Reports in colonial documents and travel reports from the early 20th century indicate that tungiasis caused severe morbidity among indigenous populations such as grave inflammation in the feet, deep ulcers, gangrene, lymphangitis, and septicaemia [1]. The suffering was so intense that affected individuals cut off their inflamed toes in sheer desperation. It is also reported that military operations sometimes had to be stopped because the feet of the indigenous soldiers, who did not wear shoes, were so sore and mutilated that they could not walk [8], [9]. Decle reported in 1898:

“… I was met by a man who came to show me his foot. The little toe was enormously swollen and full of matter (pus). I dressed it, and in a few moments a dozen others had collected, with their feet in an awful condition from the jiggers. Half of them had removed the parts attacked, cutting themselves to the bone. All these sores were most dreadful, and all I would do was to dress them.”

Decle also noted that tungiasis perpetuated poverty:

“….In some villages of Uduhu, I found the people starving, as they were so rotten with ulcers from jiggers that they had been unable to work in their fields, and could not even go to cut the few bananas that had been growing.”

Mutilation of the feet was so common that it caused a characteristic gait alteration:

“A knowing eye may always perceive when the feet of negros are the abode of the chigoe [chigoe = jiggers; designation of tungiasis in the Caribbean]. They dare not place their feet firmly on the ground, on account of the pain such a position would give them, but they hobble along with their toes turned up. And by this you know that they are not suffering from tubboes [local designation of yaws], but from the actual depredations of the chigoes.” [10].

## Biology and Transmission of an Intriguing Parasite

*Tunga penetrans* and *Tunga trimamillata* belong to the genus Tunga of the order Siphonaptera and are unique within the realm of fleas in such a way that nonfertilized females penetrate into the skin and remain there until they die in situ after 4 to 6 weeks [11], [12]. By its last abdominal segments, which form a kind of cone, the parasite remains in contact with the environment to breathe, defecate, copulate, and expel eggs, offering an opening of 250 to 500 µm in the skin as a possible entry point for pathogenic microorganisms [13].

The off-host part of the sand flea cycle is similar to other Siphonaptera species. Expelled eggs fall to the ground and develop into larvae, pupae, and adults in the immediate surroundings. Larvae hatch after 1 to 6 days (mean 3–4 days) and pupation takes place after another 5–7 days [12]. The formation of adult fleas inside the puparium needs 9–15 days [12]. Under favorable conditions, an adult sand flea will emerge about 20 days after an egg has “touched down” [14].

**Figure 1:**
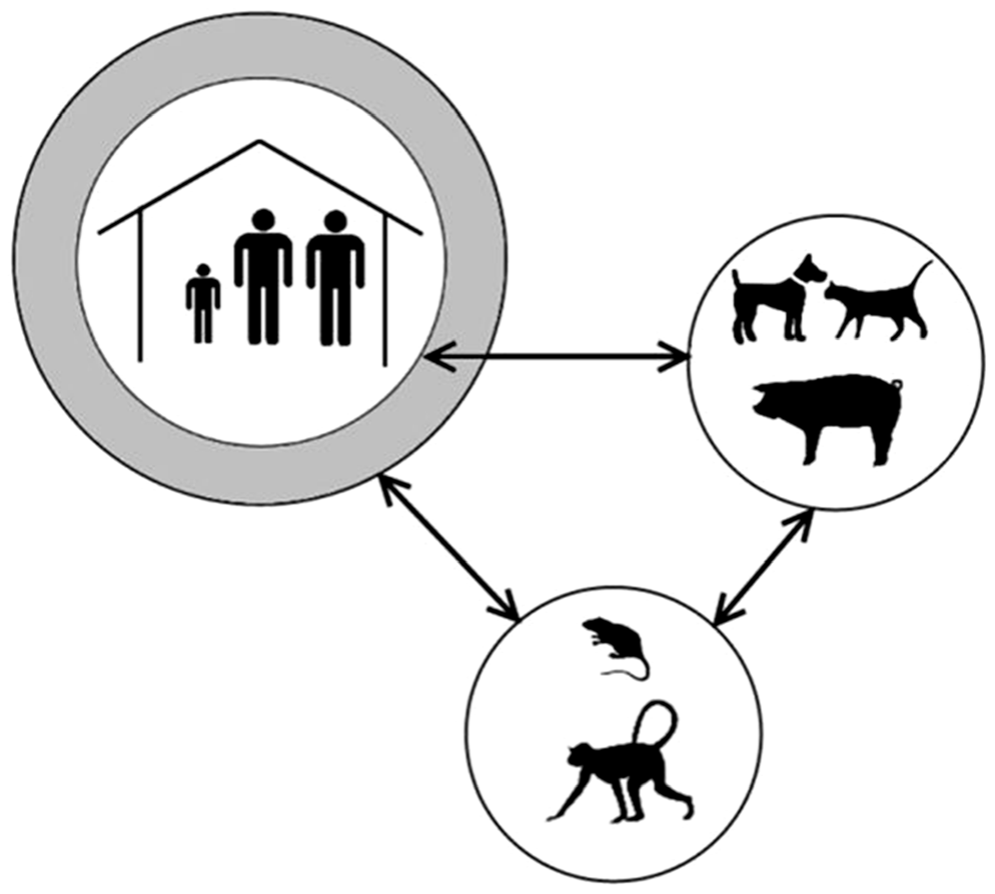
Life cycle of *T. penetrans* in a tropical environment. The human, domestic, and sylvatic cycles are linked but without close overlap.

**Figure 2:**
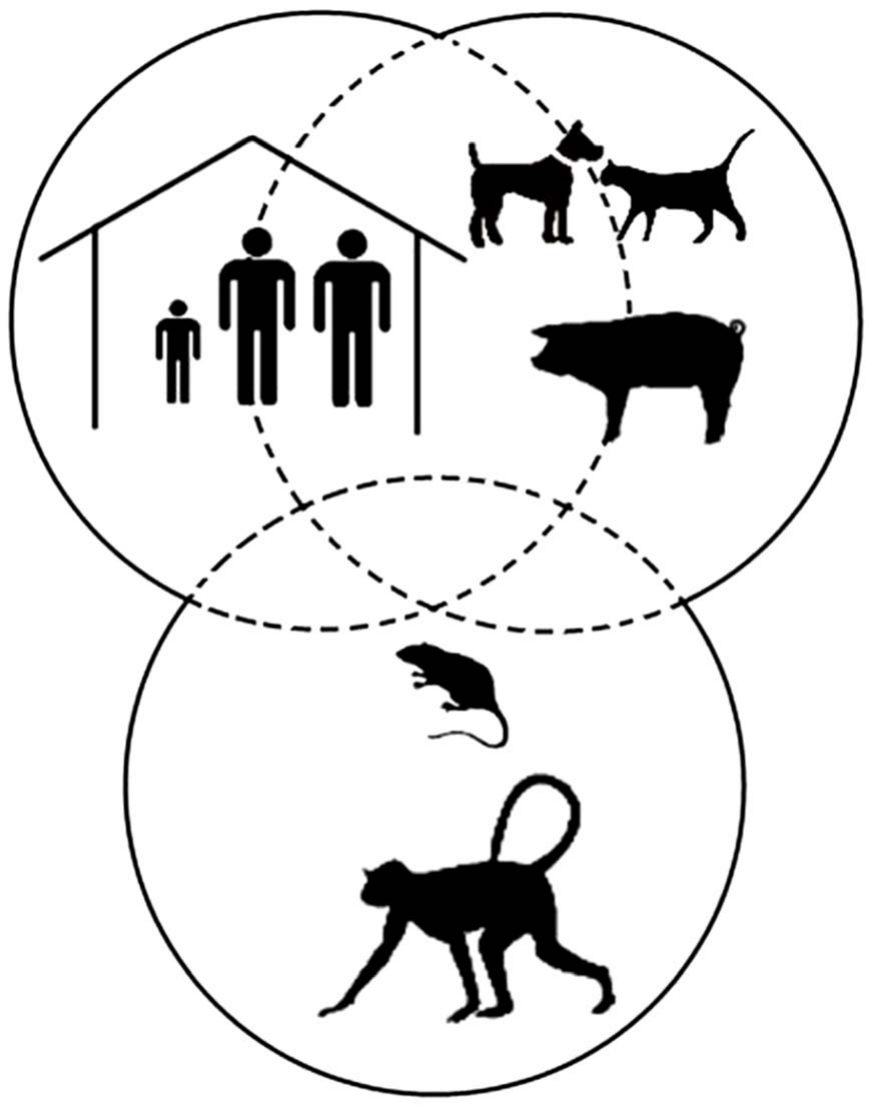
Life cycle of *T. penetrans* in a tropical environment. The human, domestic, and sylvatic cycles overlap closely.

Three life cycles of the sand flea coexist in a tropical environment: a human, a domestic animal, and a sylvatic cycle (Figure 1). These cycles overlap, partially or totally, depending on the context (Figure 2). In rural South America, e.g., dogs and cats will be around in and out of the house during the day, whereas small rodents enter during the night. In Uganda, pigs, sheep, and goats inhabit the house with their owners during the night to prevent them from being stolen. Local patterns of cohabitation between humans and animals thus explain why different animal species will act as reservoirs for tungiasis in different settings [15]–[17].

In dwellings without a solid floor—common in resource-poor settings in South America and sub-Saharan Africa—the sand flea's whole life cycle may be completed indoors [14]. *T. penetrans* is one of the few parasites that can maintain its whole life cycle in a person's sleeping quarter. Eggs expelled when a person sleeps will fall directly down to the floor or fall later, when the bed is made. The eggs may then be transferred to crevices and holes when the floor is swept. The larvae feed on the ever-present organic material. Eventually, adults emerging from pupae adhere to and penetrate into the skin, when a person places his or her naked feet on the ground. If people have to sleep on the floor, sand fleas will also penetrate parts of the body other than the feet. Ectopic penetration sites are common in the poorest of the poor, in those who do not even have a bed [18].

Similarly, classrooms in rural Africa with floors containing holes, cracks, and flaked mortar constitute ideal breeding and transmission places. Children, who often do not wear closed shoes, put their feet (many hosting a dozen viable sand fleas) on the ground, exposing their skin for many hours (Hermann Feldmeier, unpublished observation, 2013). The number of feet is high in a classroom, which means many potential carriers of the parasite and also a substantial amount of organic material on the ground.

## The Pathogenesis Explains the Morbidity and the Consequences for Public Health

The inflammatory response around burrowed viable, dead, or decaying sand fleas is the basis for the clinical and pathological manifestations. Acute inflammation—characterized by erythema, edema, pain, and itching—is caused by the growth of a biologically active foreign body within the epidermis, exerting pressure on the surrounding tissue [11]. A bacterial superinfection will increase the inflammatory response. In endemic areas, this is almost always the case. Aerobic and anaerobic bacteria (Clostridia included) have been isolated from embedded sand fleas [13]. It is not far-fetched to assume that bacteria present on the ground may adhere to adult fleas. The bacteria are then carried into the host's epidermis, eventually reaching the dermis when the parasite penetrates the basal membrane of the epidermis and inserts its proboscis into dermal capillaries. Pathogens could, of course, also be actively introduced underneath the skin through scratching [13].

Another inflammatory pathway seems to be related to the presence of endosymbiotic Wolbachia bacteria regularly present in burrowed sand fleas [19]. When the parasite dies in situ, Wolbachia-derived lipopolysaccharides are released in the surrounding tissue, which will result in an inflammatory response.

In resource-poor settings with a low coverage of tetanus vaccination, circumstantial evidence even points to a causal relationship between tungiasis and tetanus [20]–[22]. This is not surprising; *Clostridium tetani* is a soil pathogen. Adult sand fleas may passively pick up spores of the bacteria and carry them into the human skin. Alternatively, scratching of the lesion with soil-contaminated fingers may introduce the pathogen in a lesion.

These various patho-physiological mechanisms may cause suppuration, abscess formation, ulcers, lymphangitis, tissue necrosis, and gangrene. The result will be chronic pain, disability, and disfigurement, ending in mutilated feet responsible for the characteristic gait seen in individuals living in endemic areas [10]. In Brazil, all heavily infected children were observed to have walking difficulties [23]. Children with tungiasis are said to have disproportionately high absenteeism from school and to perform worse in school than unaffected pupils, since constant itching and pain make it difficult to concentrate [24].

The chronic sequels are debilitating and incapacitating [25]–[27]. Disfigurement and mutilation of the feet will impair mobility, hinder normal day-to-day activities, and have a detrimental effect on household economics, which are much dependent on the physical fitness of the adult household members.

The pathogenesis of the chronic manifestations of tungiasis, such as desquamation, hyperkeratosis, formation of fissures and ulcers, hypertrophy of nail rims, deformation of toes, and deformation and loss of nails, is presently not understood.

In an act of desperation, affected individuals often try to get rid of the parasites by using sharp instruments such as safety pins, needles, scissors, a knife, a thorn, or a sharply pointed piece of wood. These instruments are usually not disinfected and are also used by and for several household members and neighbors (Hermann Feldmeier, unpublished observation, 2013). Since a burrowed sand flea cannot be extracted without causing hemorrhage, this traditional treatment of tungiasis has to be considered as a possible way to transmit blood-borne pathogens such as hepatitis B and C virus and, perhaps, also HIV, from one person to another. Theoretically, the extremely high prevalence of hepatitis B virus infection in children in many countries in sub-Saharan Africa could be partially attributed to this treatment of tungiasis [28], [29].

Severely inflamed toes and mutilated feet cannot be hidden. Individuals with sand flea disease feel ashamed as can be seen in other parasitic skin diseases with abhorrently inflamed skin [30]. Children with tungiasis are teased and ridiculed in rural Kenya [24]. In Brazil and Nigeria, patients with tungiasis suffer from social stigmatization [4].

## There Are No Reliable Data on Disease Occurrence

The broad array of symptoms and the social stigma associated with tungiasis do not make the burden of disease easy to assess. Reliable data on disease occurrence are available neither at national nor at regional levels. Tungiasis has a heterogeneous distribution; most commonly and most severely affected are resource-poor strata of tropical and subtropical populations [23], [28].

It is, however, known that *T. penetrans* is widespread in South America and sub-Saharan Africa, and that it occurs on several Caribbean islands [25], [31]–[36]. In South America, tungiasis is known in all countries with the exception of Chile. In sub-Saharan Africa, all countries including Madagascar and the Comoro Islands seem to be affected. As a rule of thumb, tungiasis thrives when living conditions are precarious, such as in poor villages located near the beach, in rural communities in the hinterland, in the periphery of small towns, and in slums of big cities [4], [25], [8], [37], [38].

The incidence follows a distinct seasonal variation with peak transmission in the dry season [39]. During the high transmission season, the prevalence in resource-poor rural and urban communities in Brazil, Nigeria, and Madagascar may be up to 60% [4], [31]–[33], [35], [36], [40]. Prevalence, intensity of infection, and morbidity are closely related [41]. Reports in printed and electronic lay media indicate that tungiasis has re-emerged in recent years in East Africa with several hundred thousand cases in Uganda alone, of which many were associated with intense morbidity [42], [43].

Tungiasis is an important emerging infection in travelers returning from endemic areas in South America and sub-Saharan Africa [44]. In travelers the morbidity is usually low [45], [46].

Attack rates vary from setting to setting and may be as high as six newly penetrating sand fleas per individual per 24 hours. In a slum in Northeast Brazil, all 47 individuals returning to their households from a non-endemic area became infected within three weeks [47]. The age-specific prevalence shows a characteristic s-shaped pattern with a maximum prevalence in children between 5 and 14 years and elderly people [34]. Such an age-specific prevalence curve may indicate that there is no development of a protective immunity against penetrating sand fleas.

The question arises, why has sand flea disease re-emerged in such a dramatic way? The explanation for this most probably lies in the complex interactions between the parasite and the impoverished segments of various populations. After its introduction to the African continent, tungiasis had been confined to people in the rural hinterland [2]. With the construction of roads and an increasing mobility, the parasite could easily extend its spatial distribution: if an infested individual does not wear closed shoes and boards a bus or a pick-up taxi, expelled eggs will fall on the floor of the vehicle and contaminate the soil when the floor of the vehicle is cleaned out at its destination. Hence, today *T. penetrans* might easily travel hundreds of kilometers a day. Since the off-host life cycle can take place wherever the soil is appropriate and suitable animal hosts occur, the local propagation starts easily [28].

There is a general agreement that the occurrence of tungiasis is linked to poverty [37], [38]. Poverty is in fact such a constant characteristic of sand flea disease that the prevalence of tungiasis can be considered as a proxy for the economic development of a community. Occurrence data of tungiasis are probably a better description of what is going on in a community than economic averages and public health surveillance indices [48].

We are convinced that the presence of a dozen or more viable sand fleas in the feet of a child —a clear proxy of repeated and untreated infections—is a compelling indicator of inattention to children's needs. A survey in a secondary school in the Busoga subregion, north of Lake Victoria, Uganda, showed that less than 5% of the mothers regularly inspected the feet of their children and attempted to remove embedded fleas (Hermann Feldmeier, unpublished observation, 2013). Regular inspection of the feet and removal of penetrated sand fleas are, for now, the optimal methods to keep clinical pathology at bay.

## Morbidity Control Is Feasible

Sand flea disease is a zoonosis affecting a broad spectrum of domestic and sylvatic animals; hence, control can only be achieved by a trans-disciplinary approach (Table 1). The “One Health” concept should be a suitable framework for this, since it means that improvements of human and animal health are aimed for simultaneously [49]. A small field trial in Brazil showed that a combination of 10% imidacloprid and 50% permethrin temporarily reduced the parasite load [50].

**Table 1:** Major goals and needs for achievements and control of tungiasis.

Goals	Needs
1. Assess the burden of disease and define regions and population groups that will most benefit from effective control measures	• Perform systematic prevalence and morbidity studies in all countries in which tungiasis occurs or is supposed to occur
	• Establish geographical distribution of tungiasis in humans and animals in the Americas and in sub-Saharan Africa
2. Understand the role which animal reservoirs play and describe characteristics of local transmission dynamics (where, when and why people get infected)	• Determine the animal reservoir in different settings
	• Design and implement methods of cooperation between the academic, the public health, and the veterinary sectors
	• Determine the duration of the transmission season(s) according to the climate characteristics
3. Assess the economic and societal impact of tungiasis in impoverished settings	• Establish animal models of tungiasis to evaluate the impact on livestock productivity
	• Determine the impact tungiasis in humans and animals has on household economics, performance in school, and access to local health and administrative infrastructure
	• Quantify life quality impairment in patients with tungiasis
4. Eliminate health risks associated with the neglect of acute and chronic tungiasis-associated morbidity and the treatment with inappropriate instruments	• Identify pathogens transmitted through inappropriate treatment with sharp instruments
	• Provide simple, safe, effective, and sustainable means for self-treatment of tungiasis
	• Screen locally available plants for repellent activity against sand fleas
	• Prevent tungiasis through elimination of breeding sites and animal reservoirs
5. Raise awareness and create intersectional cooperation based on “One Health” principles	• Perform information, education, and communication campaigns—work on eliminating the stigmatization associated with tungiasis (“It is nothing to be ashamed about!”)
	• Create a community-led demand for community-based interventions: let local communities speak for themselves
	• Take advantage of flea control approaches in use for domestic animals
	• Identify stakeholders on all levels from global to regional and from national to local community

**Figure 3:**
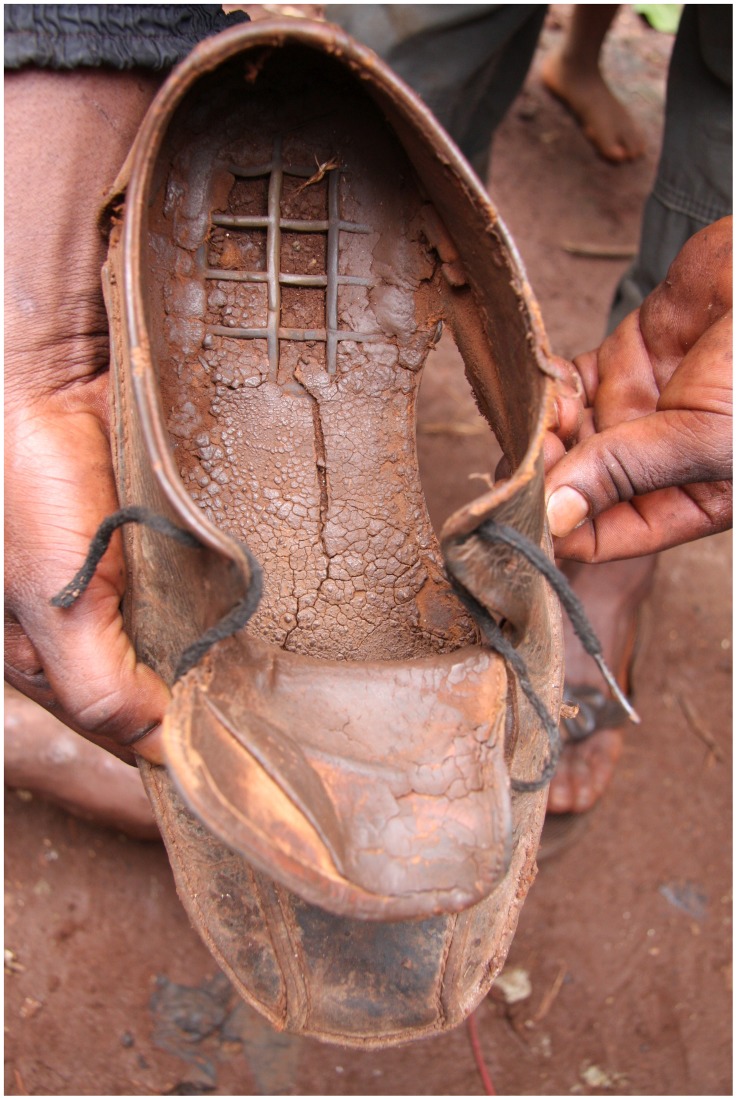
Shoe of an adolescent from rural Kenya after 6 months of use.

Up to now, no drug treatment has been found to be effective against burrowed sand fleas in humans [51], [52]. Prevention is therefore the only available means to control morbidity. Closed, solid shoes partially protect against invading sand fleas [53]. Confronted by the reality of resource-poor communities, however, protection by shoes remains theoretical. Firstly, exposure frequently takes place inside houses, where shoes usually are not worn. Secondly, shoes are considered as valuable assets and will often not be used in schools, for walks, or playing around the house. Thirdly, shoes have a tendency to perish rapidly, and sand fleas can easily reach the skin through cracks and holes (Figure 3).

A more realistic preventive approach is possible by application of a repellent based on coconut oil (Zanzarin). Studies in Brazil and Madagascar show that a twice-daily application of this repellent to the feet could reduce the attack rate by almost 100 percent [41], [53]–[55]. Existing tungiasis-associated morbidity was resolved within a couple of weeks and mobility was regained. From these studies one could conclude that acute—and to a lesser extent also chronic—pathology is reversible, if new penetrations are prevented.

## Final Considerations

Tungiasis has an important social dimension and affects human rights. Being a zoonosis and considering its intricate links with poverty, it requires trans-disciplinary research in which e.g., public health, social sciences, health education, and animal husbandry need to interact. Obviously, tungiasis has the potential to trigger political anti-poverty strategies by simultaneously addressing public health infrastructures in both humans and animals at the community level.

Based on general characteristics used by WHO to determine which diseases are categorized among the NTDs, there are compelling reasons for the inclusion of tungiasis in this group. All countries where tungiasis is reported are low-income and lower middle-income economies where this plague afflicts the same marginalized population segments and communities affected by the already-recognized NTDs. The same inequity issues and complex social determinants as in the field of the NTDs have perpetuated the transmission of tungiasis in resource-poor communities in South America and sub-Saharan Africa. The recent evidence of effective intervention measures against tungiasis should no longer be ignored by global health organizations. WHO, through its regional offices for the Americas and Africa, should work to raise awareness among NTD stakeholders and formulate appropriate strategies to address this debilitating and mutilating parasitic skin disease that has unnecessarily plagued disadvantaged communities for centuries.
